# Investigating the structural properties and wear resistance of martensitic stainless steels

**DOI:** 10.1371/journal.pone.0312242

**Published:** 2024-11-19

**Authors:** Alok Bhadauria, K. Venkateswara Reddy, Rajesh K. Khatirkar, Din Bandhu, Prashant Kumar Gangwar

**Affiliations:** 1 Department of Mechanical and Industrial Engineering, Manipal Institute of Technology Bengaluru, Manipal Academy of Higher Education, Manipal, Karnataka, India; 2 Department of Mechanical Engineering, Marri Laxman Reddy Institute of Technology and Management, Hyderabad, Telangana, India; 3 Department of Metallurgical and Materials Engineering, Visvesvaraya National Institute of Technology (VNIT), Nagpur, Maharashtra, India; 4 Department of Construction Technology & Management, Woldia University, Woldia City, Ethiopia; Western Carolina University, UNITED STATES OF AMERICA

## Abstract

The present work explores the microstructures and abrasive wear behavior of AISI 410 and AISI 420 martensitic stainless steels after hardening and tempering. Microstructural changes and wear were analyzed using optical microscopy and SEM. Different heat treatments resulted in varying hardness values, with a slight increase at 723 K due to (Fe,Cr)_23_C_6_ formation, and a significant reduction at 873 K. SEM and EDS showed AISI 410 had a martensitic structure without notable precipitates, while AISI 420 exhibited coarser and new carbide precipitations after tempering. XRD confirmed martensitic peaks and carbide formation (Cr₃C₂, Mo₂C), improving wear resistance through carbon and chromium segregation. No direct correlation between bulk hardness and abrasive wear resistance was found. AISI 410 showed lower wear mass than AISI 420, with wear mechanisms including micro-cracking, ploughing, groove formation, and particle pullout. Wear debris consisted of machining chips and flaky particles, offering insights into the wear processes.

## 1. Introduction

The capability of martensitic stainless steels (MSSs) to be hardened and tempered makes them suitable for applications like shafts, valve bodies, fasteners, coal handling and mining equipment, spindles requiring corrosion resistance and heat resistance with a combination of good strength and fatigue properties [1, 2]. MSSs contain approximately 11–12% chromium which provides good corrosion resistance [3]. These types of steel can become martensitic when quenching done in water or oil from austenitizing temperature [4]. Martensite is brittle in its quenched state, so it undergoes tempering to achieve an appropriate balance of hardness, ductility, and toughness [5, 6]. MSSs are hardened by austenitizing in the temperature range 925°C to 1065°C followed by oil quenching. The austenitizing temperature is selected to achieve the desired level of carbide dissolution while avoiding overheating, which could result in ferrite formation [7, 8]. Higher austenitizing temperatures promote greater carbide dissolution, improving both corrosion resistance and strength. The hardness and strength of martensitic stainless steels (MSSs) remain stable in the as-quenched condition up to a tempering temperature of 450°C, beyond which they decline sharply [9, 10]. Elevated austenitizing temperatures result in more chromium dissolution, sharpening the secondary hardening peak in specimens treated at these higher temperatures. Temper embrittlement occurs between tempering temperature of 425°C and 565°C, so tempering within this range should be avoided for applications sensitive to impact [11–13]. The martensitic microstructure is very rarely used in its untempered state owing to the substantial internal stresses generated by the martensitic phase transformation, along with its very low ductility and toughness [14, 15]. Tempering at low temperature effectively alleviates these internal stresses without any substantially altering the fundamental characteristics of martensitic microstructure [16, 17]. On the contrary, tempering at higher temperature modifies the internal structure, thereby improving ductility and toughness [18]. Lee at al. [19] observed change in microstructure and mechanical performance of AISI 4340 steel under different quench and tempered conditions. The result reveals that varying the tempering temperature and duration has a significant impact on both the microstructure and mechanical properties. The strength and hardness of tempered martensite decrease with increasing in tempering temperatures and prolonged holding times. Conversely, ductility generally improves with increasing tempering temperature and duration, except in cases where tempered martensite embrittlement occurs. Mirzaee et al. [10] studied the effect of tempering temperature (250–650°C) on mechanical proprieties and microstructure of 410 and 410 Ni MSSs and observed that 410 Ni showed improved ductility and fracture toughness compared to 410 owing to Ni’s influence on stacking fault energy. The ductile fracture was observed at 250°C in both samples. However, at tempering temperatures of 450–650°C, the 410 Ni sample exhibited intergranular brittle fracture, while the 410 sample showed brittle fracture. Jiang et al. [20] investigated the effect of tempering time and temperature on the microstructure and properties of martensitic stainless steel and observed that tempering below 300°C prevents major carbide precipitation, which is vital for corrosion resistance. However, tempering around 550°C maximizes chromium-rich carbide precipitation, reducing corrosion resistance. They concluded that the 400 to 600°C range as crucial for balancing mechanical properties and corrosion resistance. In another work, Xiong et al. [21] reported that thermal cyclic heat treatment and tempering enhance strength and toughness of MSSs by refining grain size from 20 μm to 5 μm and improving cryogenic toughness from 30 J to 60 J at -196°C and reducing the ductile–brittle transition temperature.

In many applications employing MSSs, sufficient wear resistance is essential alongside the strength, ductility, and toughness of the steels. There are generally two types of abrasive wear: (i) two body abrasion and (ii) three body abrasion. A material’s hardness, as well as the form, size, quantity, and distribution of second phase particles, as well as the characteristics of abrasive particles all affect resistance of abrasive wear [22, 23]. Puli et al. [24] produced AISI 410 MSS coatings on low-carbon steel via friction surfacing and manual metal arc welding (MMAW). Wear resistance of friction surfaced coatings (contains fully martensite) was comparable to bulk AISI 410 steels. On the other hand, lower wear resistance and hardness were observed in MMAW coatings (contains significant amount of δ-ferrite) in comparison with friction surfaced coatings. Generally, the abrasion resistance in both ferrite/carbide and pearlite/carbide microstructures is influenced by the volume fraction of carbides [25, 26]. Abrasive resistance in microstructures with pearlite-carbide improves with increasing carbide volume percent up to approximately 35%. Microstructures with ferrite-carbide, abrasion resistance is not considerably impacted by the carbide volume fraction. In the study by Yıldız et al. [27] examined how cryogenic treatment and tempering temperature affect AISI 431 steel’s mechanical and microstructural properties. They observed that tempering temperature significantly influences wear resistance, with higher temperatures improving resistance due to microstructural refinement and carbide precipitation. The study highlights the need to optimize tempering conditions for better performance in martensitic stainless steels.

In steels with a martensitic microstructure, wear resistance is affected by both the carbon percentage and the content of martensite present. Moreover, the morphology of carbides within an austenitic/martensitic matrix also plays a significant role in abrasion resistance [28].

The present work addresses a gap in the literature by focusing on the relatively underexplored microstructural developments of martensitic stainless steels (MSSs). Specifically, it investigates how austenitizing and tempering temperatures impact the microstructural evolution in AISI 410 and AISI 420 steels. The research uniquely examines the correlation between wear properties, hardness, tempering temperature, and microstructural changes, offering new insights into the behaviour of these steels.

## 2. Experimental

### 2.1 Materials and methods

AISI 410 and AISI 420 MSSs (as rolled and initial annealed condition) were used to study microstructural developments and the wear behaviour via two body abrasive wear after hardening/tempering process. Composition of as-procured steels (rolled and annealed) was measured via optical emission spectroscopy as seen in Table 1.

**Table 1:** Chemical composition (in wt% alloying elements) of AISI 410 and AISI 420 steels.

Composition (wt%)											
Steel	C	Mn	P	S	Si	Cr	Cu	Ni	Mo	V	Ti
**AISI 410**	0.11	0.44	0.028	0.018	0.35	12.09	0.01	0.07	0.02	0.02	0.001
**AISI 420**	0.18	0.77	0.025	0.018	0.32	12.26	0.01	0.08	0.02	0.03	0.001

All the samples underwent a hardening process that involved austenitizing for 2700 seconds at 1198 K (± 3 K) and then oil quenching. Samples were cooled in air to approximately 298K after being tempered for 2700 seconds at 423, 573, 723, and 873 K temperatures, respectively, after the hardening treatment. It’s crucial to note that tempering was performed right away following the hardening procedure. Both the hardening and tempering processes were conducted in an inert atmosphere furnace to prevent oxidation during the heat treatments. Subsequently, All the samples were polished using progressively finer emery papers. Following this preliminary polishing, all samples were further polished using an Al_2_O_3_-water slurry on velvet fabric. In order to view the microstructures, they were lastly polished using diamond paste up to 0.25 μm while being lubricated with Hiffin chloride as lubricant to observe the microstructures. The mirror polished samples were then etched with Vilella’s reagent [29] to observe the microstructure. Optical microscope (ZIESS, Germany) and scanning electron microscope equipped with electron dispersive spectroscopy (EDS) (JEOL, Japan 6380A) were used to see morphology and microstructures of the samples (as-quenched, hardened and tempered). X-ray diffraction (XRD: Bruker Xflash: 30 system, Germany) was used to characterize the different phase in the samples. The bulk hardness was determined using a Rockwell hardness tester with a 150 kg load and dwell time of 10 sec. An average of four readings was taken for each sample.

### 2.2 Wear testing procedure

Pin on disc (model: TR20-LE, Ducom, India) was utilized for performing two body abrasive wear tests. The tests were carried out at room temperature. The temperature and relative humidity level were found to be consistent during all tests. The cylindrical specimens used for the two-body abrasive wear tests had dimensions of 9 mm in diameter and 20 mm in length. The pin-shaped sample’s surface was polished with emery paper (till1200 mesh) before starting the test. Samples was secured with the help of holder, however 150-grit silicon carbide abrasive paper was fixed on a rotating steel disc. After the test started, new abrasive paper was utilized every 300 seconds to prevent the fragmentation of particles. Ethyl alcohol is used to clean the samples, and weighing (accuracy: ~0.1 mg) is done before and after the test. Wear loss was calculated based on weight difference and presented as mass loss, averaged over a minimum of three tests. The impact of load on the mass loss of martensitic stainless steel was investigated at a speed of 1.885 m/s using loads of 10N and 30N. SEM was used to analyze wear surfaces and wear debris particles to study surface morphology and abrasive wear mechanisms.

## 3. Results and discussions

### 3.1 Hardness and microstructures

**Fig 1:**
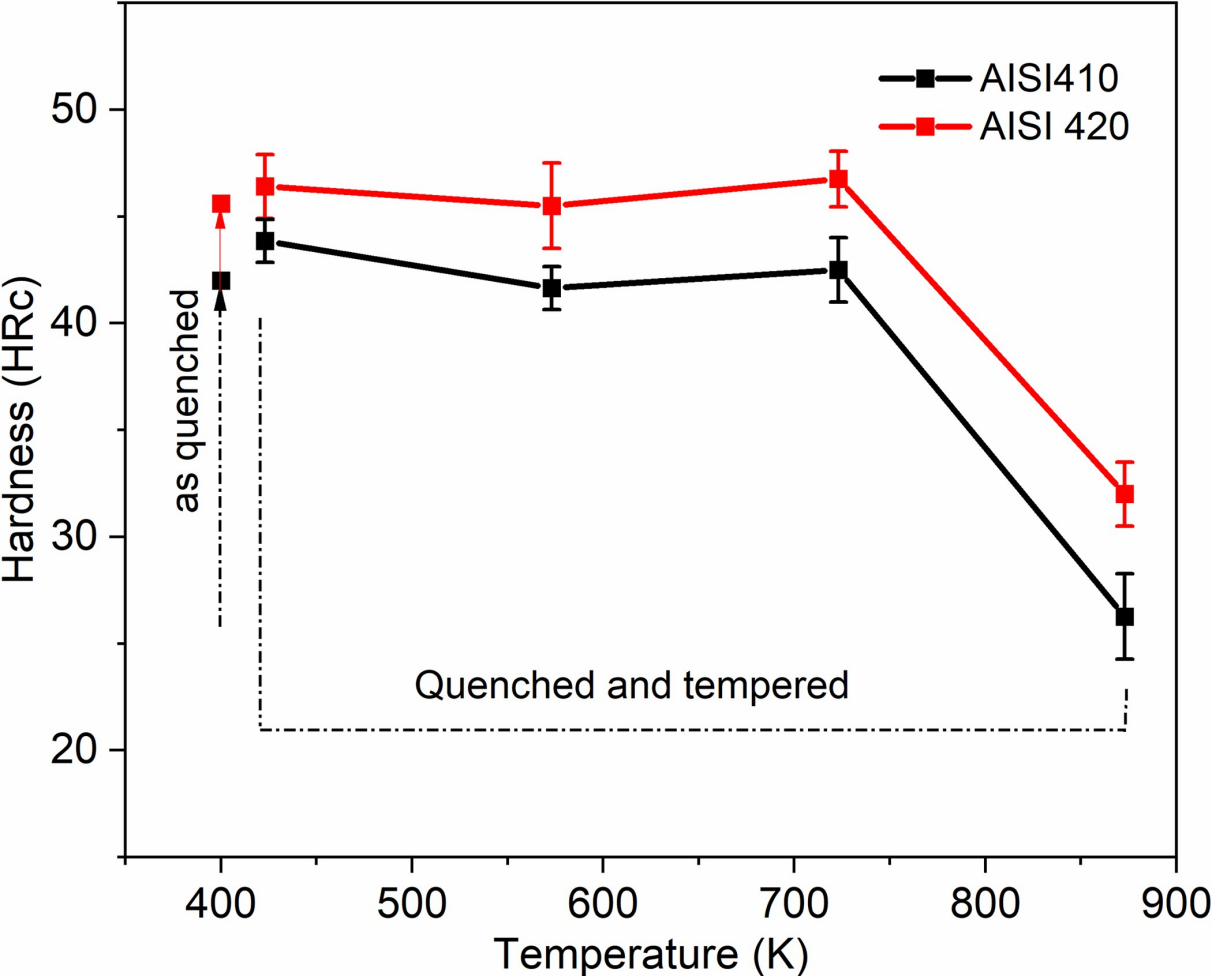
Shows the change in the hardness (HRc) with the tempering temperature.

Fig 1 represents the hardness of as quenched MSS and quenched+tempered MSS for both the steel (AISI 410, 420) with respect to tempering temperature. The hardest sample is the one that was tempered at 723 K (450°C), while the softest sample is the one that was tempered at 873 K (600°C). The drop in hardness was insignificant when tempered at 423 K (150°C), also at approximately 723 K (450°C) secondary hardening occur due to the formation of (Fe,Cr) type carbides.

**Fig 2:**
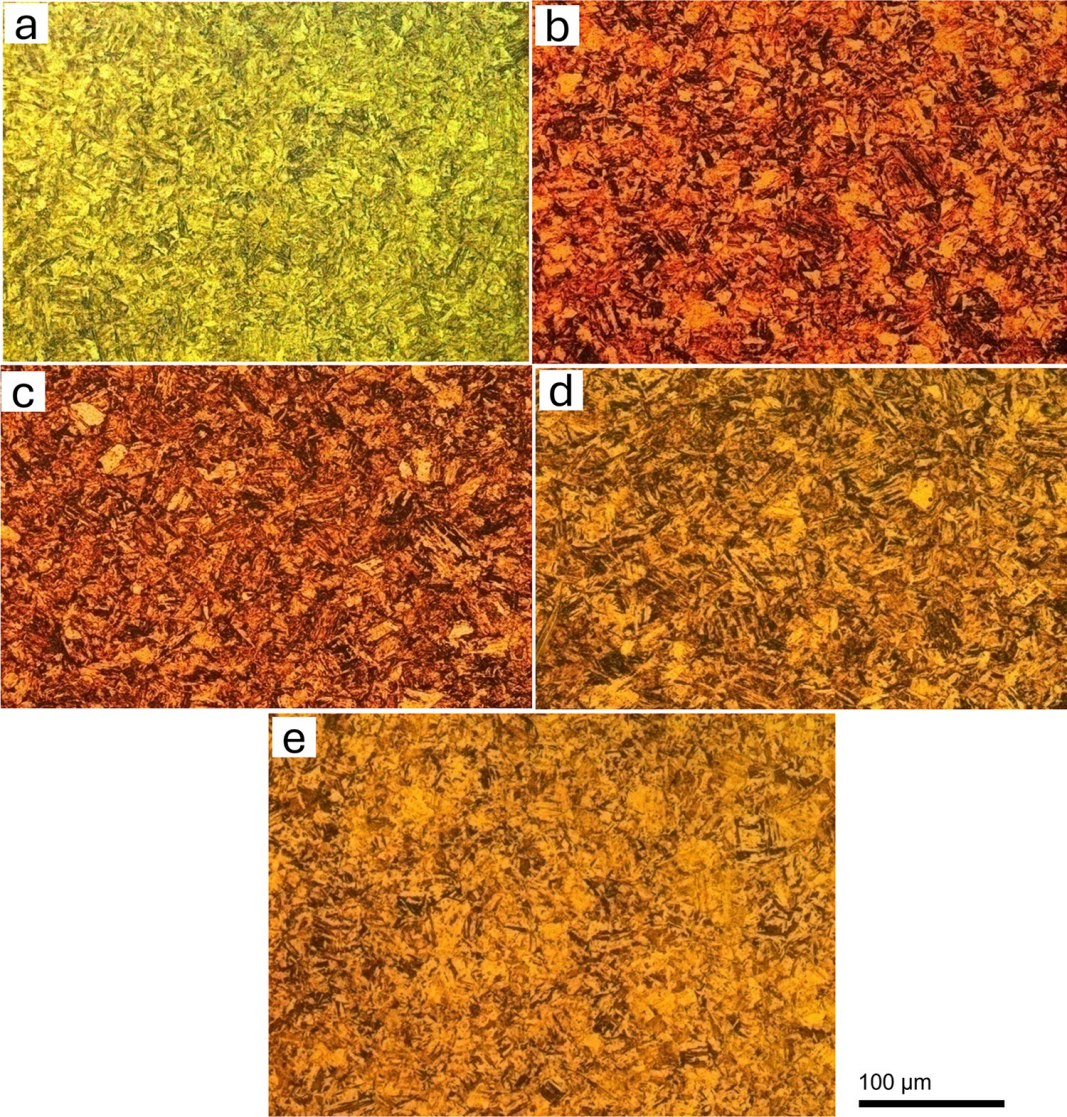
Optical microscopy images of AISI 410 in (a) as-quenched (1198K for 45 min followed by quenching in oil) condition and hardening followed by tempering at (b) 423K (c) 573K (d) 723K (e) 873K.

**Fig 3:**
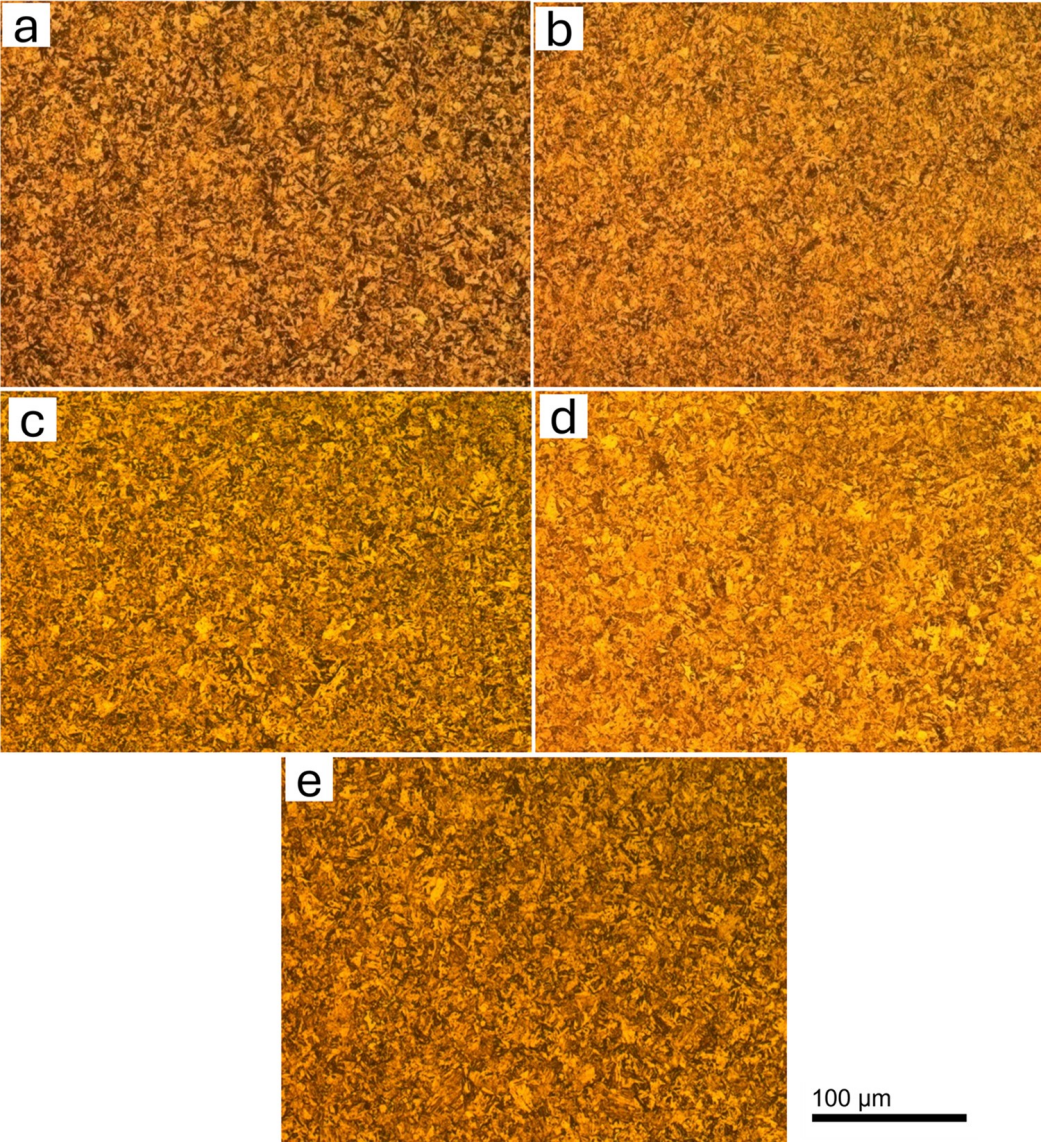
Optical microscopy images of AISI 420 in (a) as-quenched (1198K for 45 min followed by quenching in oil) condition and hardening followed by tempering at (b) 423K (c) 573K (d) 723K (e) 873K.

Previous research has shown that a microstructure consisting of martensite and Fe and Cr carbides is produced by quenching below the austenitization temperature. M_3_C is present at 723 K, but it is not present at tempering temperatures at approximately 923 K. M_23_C_6_ develops at around 723 K, and at 813 K and above, it becomes the predominant carbide. Similarly, M_7_C_3_ forms at approximately 753 K, but its quantity decreases with increasing temperatures due to its detrimental effect on corrosion resistance. This occurs during tempering at temperatures ranging from 753 K to 923 K. Optical microstructures of as quenched MSS and quenched + tempered MSS samples of AISI 410 and AISI 420 steels are shown in Figs 2 and 3, respectively.

**Fig 4:**
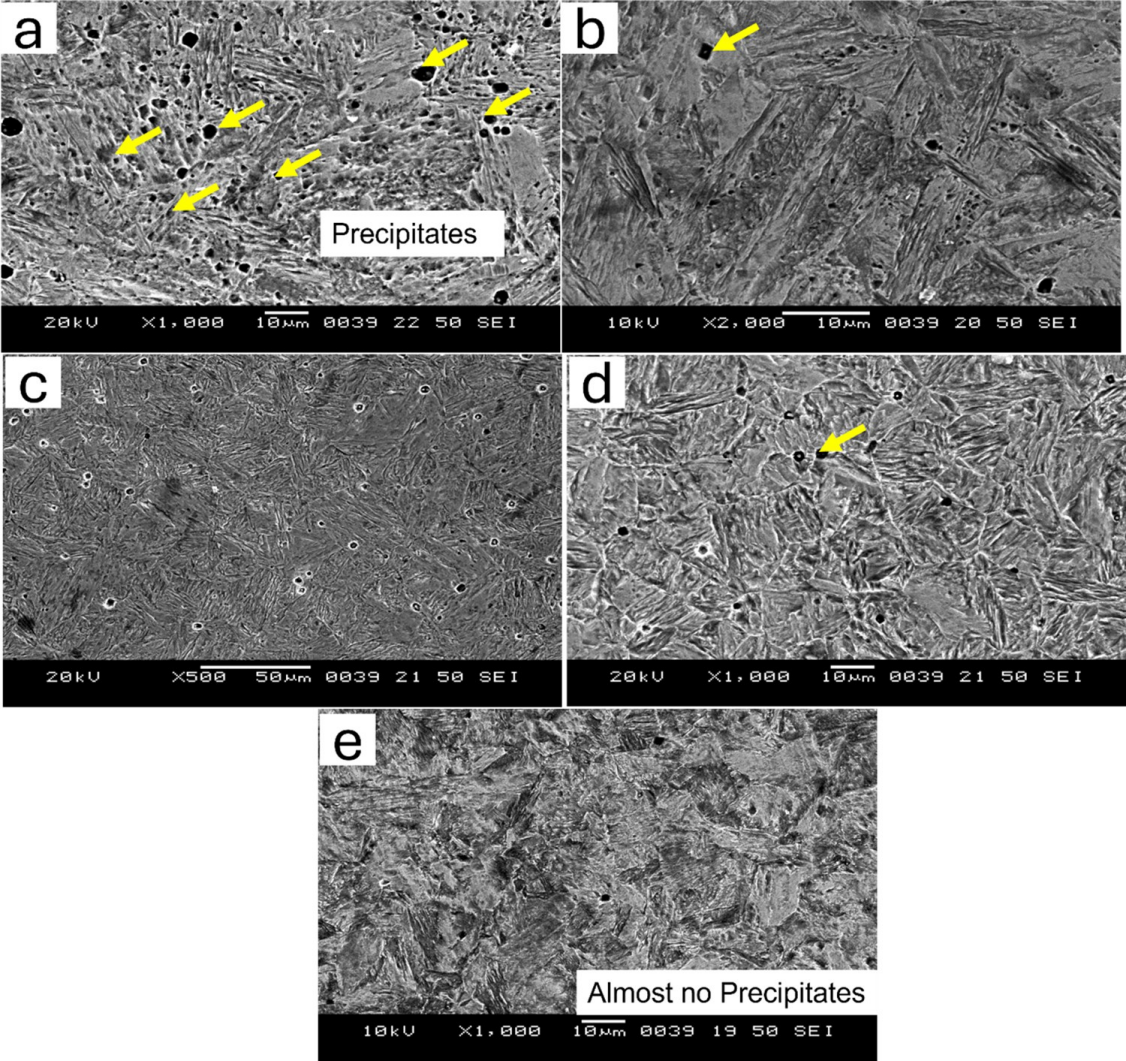
Secondary electron SEM images of AISI 410 in (a) as-quenched (1198K for 45 min followed by quenching in oil) condition and hardening followed by tempering at (b) 423K (c) 573K (d) 723K (e) 873K.

**Fig 5:**
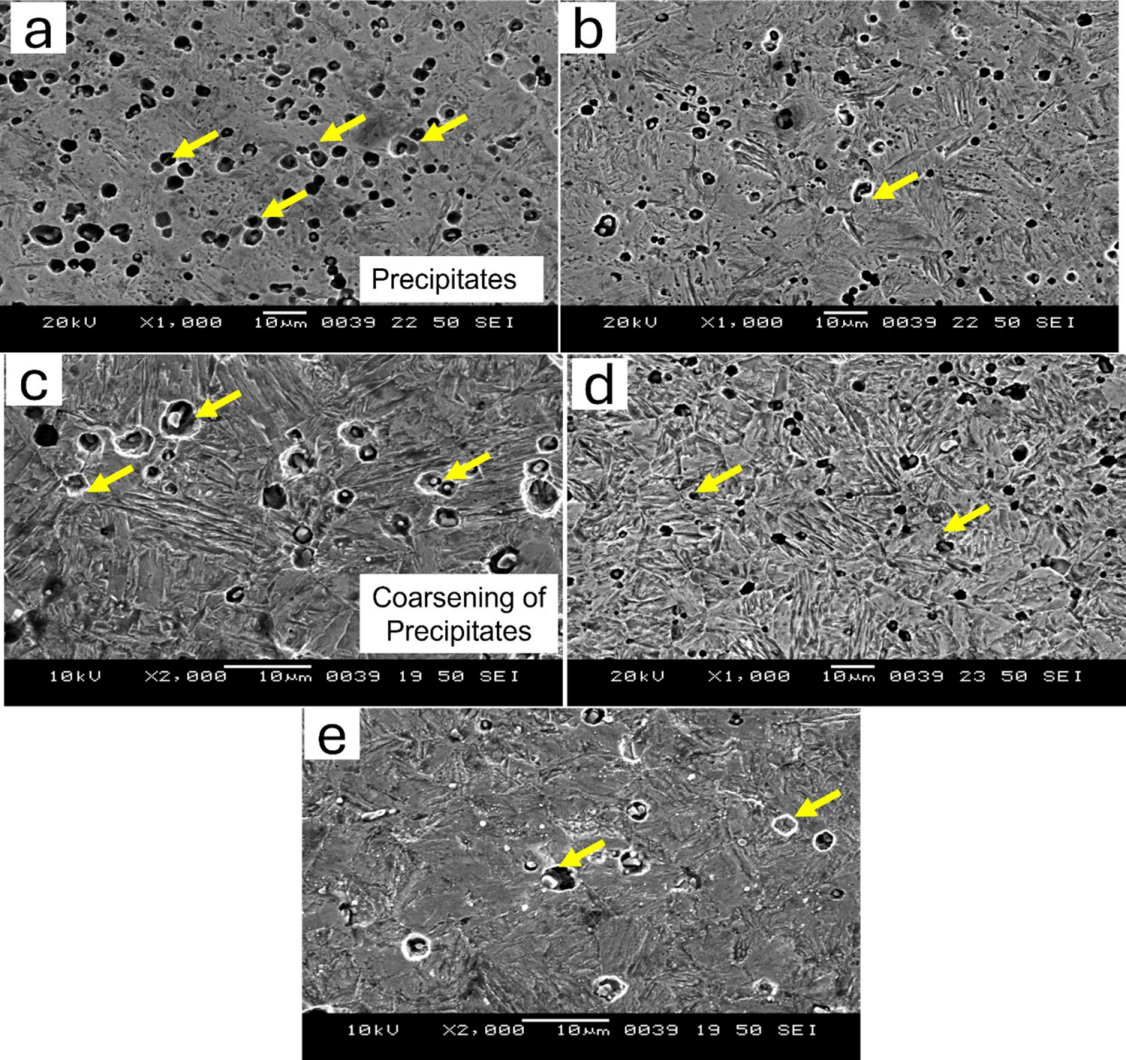
Secondary electron SEM images of AISI 420 in (a) as-quenched (1198K for 45 min followed by quenching in oil) condition and hardening followed by tempering at (b) 423K (c) 573K (d) 723K (e) 873K.

SEM of the as quenched and quenched + tempered MSS samples for AISI 410 and AISI 420 steel samples are shown in Figs 4 and 5 respectively.

It can be observed that AISI 410 martensitic stainless steel showed martensitic structure and does not contain significant amount of precipitates in the as-quenched as well as quenched+tempered condition (Figs 2 and 4). This is primarily due to the lower carbon content in AISI 410 MSS. The precipitation was not observed even after tempering at 873 K. The carbides were completely dissolved after austenitization and did not precipitate out after tempering. On the other hand, AISI 420 MSS shows the presence of undissolved carbides after austenitization. These carbides coarsened after tempering along with the precipitation of new carbides. The effect of carbon content on the carbide precipitation was clearly brought out by optical as well as scanning electron microscopy. The microstructure of AISI 420 MSS was also martensitic.

**Fig 6:**
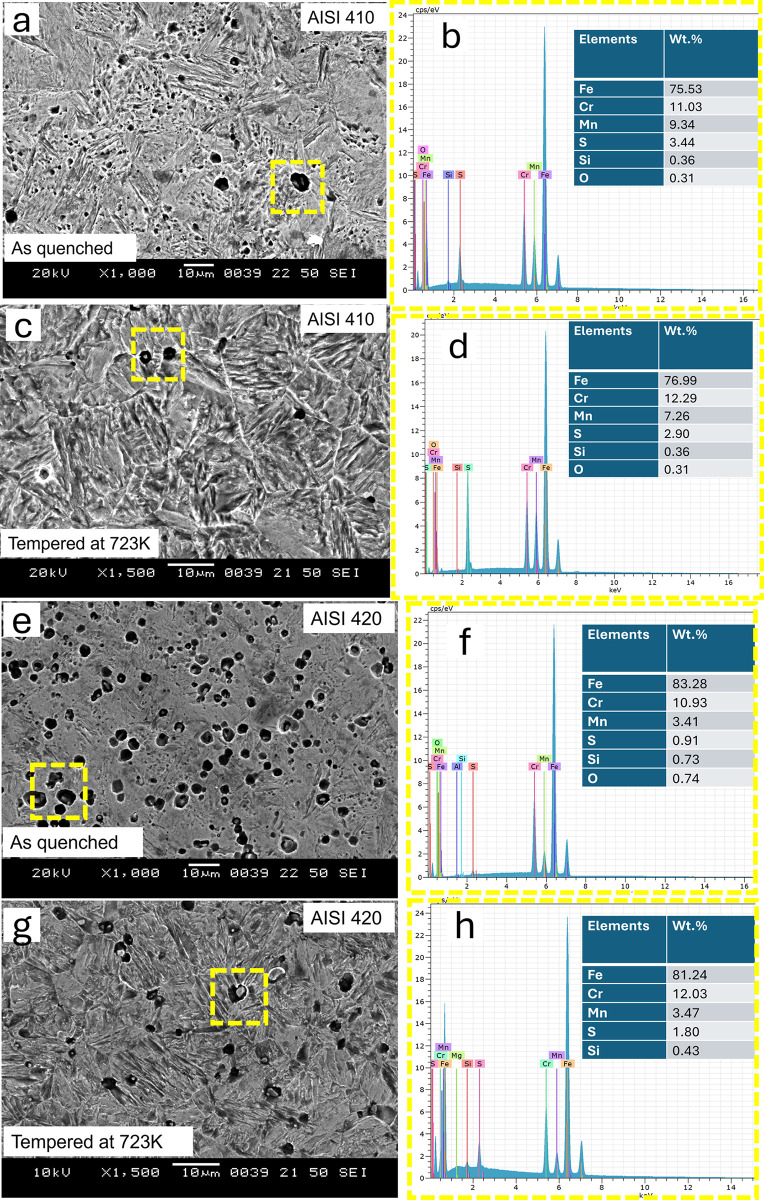
Shows the SEM images and EDS spectra of: (a), (b) as-quenched AISI 410; (c), (d) AISI 410 tempered at 723 K; (e), (f) as-quenched AISI 420; and (g), (h) AISI 420 tempered at 723 K.

Fig 6A and 6B shows the SEM images of as-quenched AISI 410 steel and the corresponding energy-dispersive spectroscopy (EDS) spectra. Fig 6C and 6D represents the SEM images of AISI 410 steel after tempering at 723 K, along with the corresponding EDS spectra. AISI 410 martensitic stainless steel exhibits a martensitic structure and contains no significant amount of precipitates in both the as-quenched and quenched-plus-tempered conditions. The EDS confirms the presence of elements Fe, Cr, Mn, S, Si, and O, which are the primary components of AISI steels. Fig 6E–6H) represents the SEM images and EDS spectra of AISI 420 steel in both as-quenched and tempered at 723 K conditions. AISI 420 martensitic stainless steel displays undissolved carbides after austenitization. These carbides become coarser following tempering, accompanied by the precipitation of new carbides. In all the EDS spectra, no significant changes are observed.

**Fig 7:**
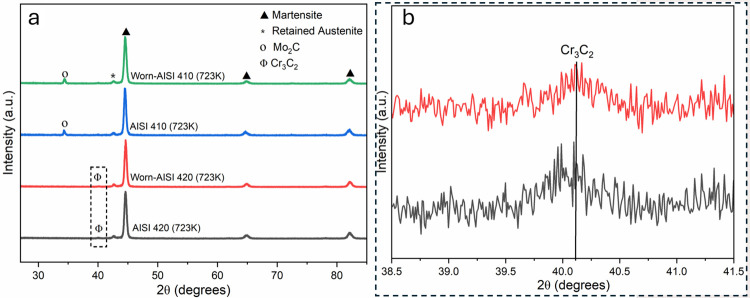
(a) X-ray diffraction patterns for AISI 410 and 420 steel after tempering at 723 K, before and after the wear test; (b) enlarged portion of the marked area at 40.04° in Fig (a).

Fig 7 shows the XRD patterns of AISI 410 and AISI 420 steel tempered at 723 K, both before and after the wear test. Prominent peaks for martensite at 45°, 65°, and 82.4° 2θ are confirmed by X-ray diffraction (XRD) analysis; these correspond to the crystallographic planes (110), (200), and (211) of the BCT structure [30]. The retained austenite exhibits FCC structure-typical diffraction peaks at around 43.5°. In AISI 420, the formation of Cr₃C₂ during tempering is attributed to the segregation of carbon and chromium, leading to carbide precipitation. The appearance of diffraction peaks near 35°, indicative of secondary carbide formation, confirms the formation of Mo₂C precipitates in AISI 410 [31]. The wear resistance of the steel increases when carbide precipitates are present.

### 3.2 Wear behaviour

**Fig 8:**
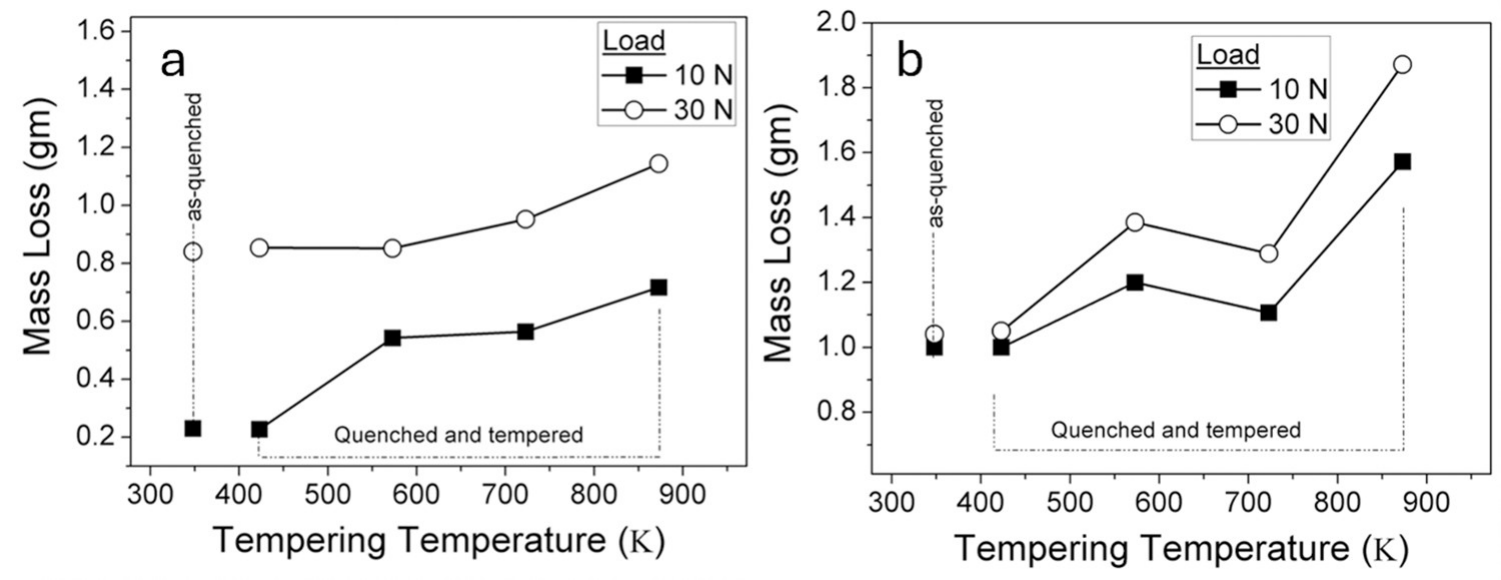
Mass loss (gm) with the tempering temperature for 10 and 30 N loads (a) AISI410, (b) AISI420, Mass loss of the as quenched sample is also represented for comparison purpose.

Fig 8 shows mass loss (in grams) in two-body abrasive wear of martensitic stainless steel under both as quenched and quenched + tempered conditions, plotted against the applied load. As quenched sample, characterized by a martensitic microstructure having undissolved iron carbides, exhibited least mass loss. In contrast, the quenched + tempered samples showed an incremental increase in mass loss as the tempering temperature increases. Fig 6a and 6b depict the influence of loads on mass loss vs tempering temperature for AISI 410 and AISI 420, respectively.

**Fig 9:**
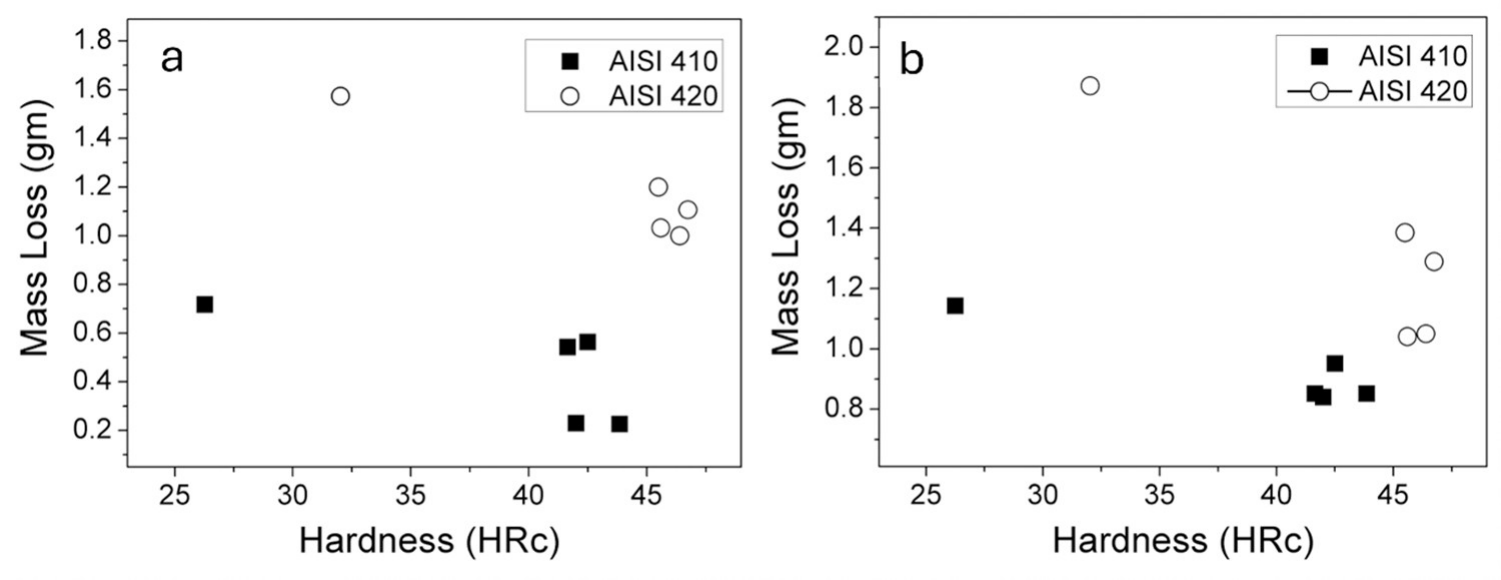
Mass loss (in gm) vs hardness (HRc) for (a) 10 N, and (b) 30 N load.

Mass loss for AISI 420 MSS samples increased gradually as the tempering temperature increases, except for 723K. As load increases from 10 N to 30 N, a comparable result has been seen observed in mass loss for all the heat treated samples. Following tempering at 723 K, the microstructure was composed of carbides (M_23_C_6_); following tempering temperature at 848 K, the carbides were M_7_C_3_. The decrease in mass loss due to wear at 723 K is associated with secondary hardening induced by carbide precipitation. The wear mass loss shows no significant dependence on hardness at both 10 and 30 N loads, as depicted in Fig 9A and 9B. However, at higher load (30 N), wear is much more noticeable—roughly 1.85 times more than at the lesser load (10 N). In the case of higher load, more abrasive particles penetrate the sample surface, which accounts for the increased wear at 30 N load. To assess the abrasive wear resistance, heat-treated samples were compared against the annealed sample as a reference. When compared to the as-received sample, the samples tempered at 423K and 573K exhibited 2.3–2.6 times greater wear resistance, however, those tempered at 873K showed 1.1–1.8 times higher wear resistance.

Zhang et al. [2] investigated how microstructure affects the friction behavior of low carbon martensitic stainless steel (MSS). In dry friction tests, quenched martensite showed the best wear resistance and lowest friction due to its high hardness. Also, High-temperature martensite showed better wear resistance than low-temperature martensite due to its superior work-hardening ability and the protective properties of reversed austenite.

**Fig 10:**
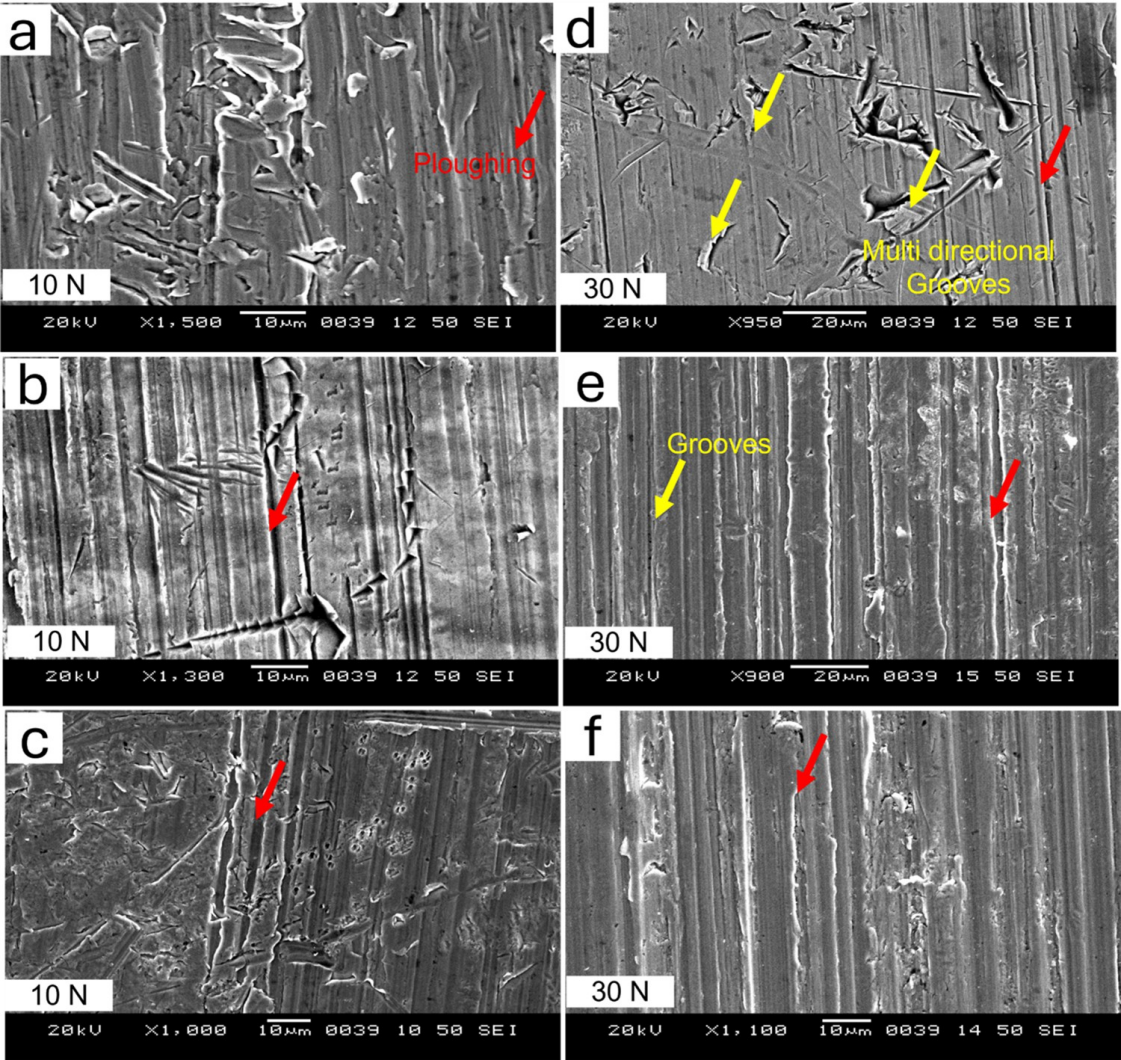
SEM images of the worn out surface of AISI410 in (a) as quenched (austenitized at 1198K for 45 min followed by quenching in oil) condition, and quenched followed by tempering at (b) 423K and (c) 873K at 10 N load, (d) as quenched, (e) 423K and (f) 873K at 30 N.

**Fig 11:**
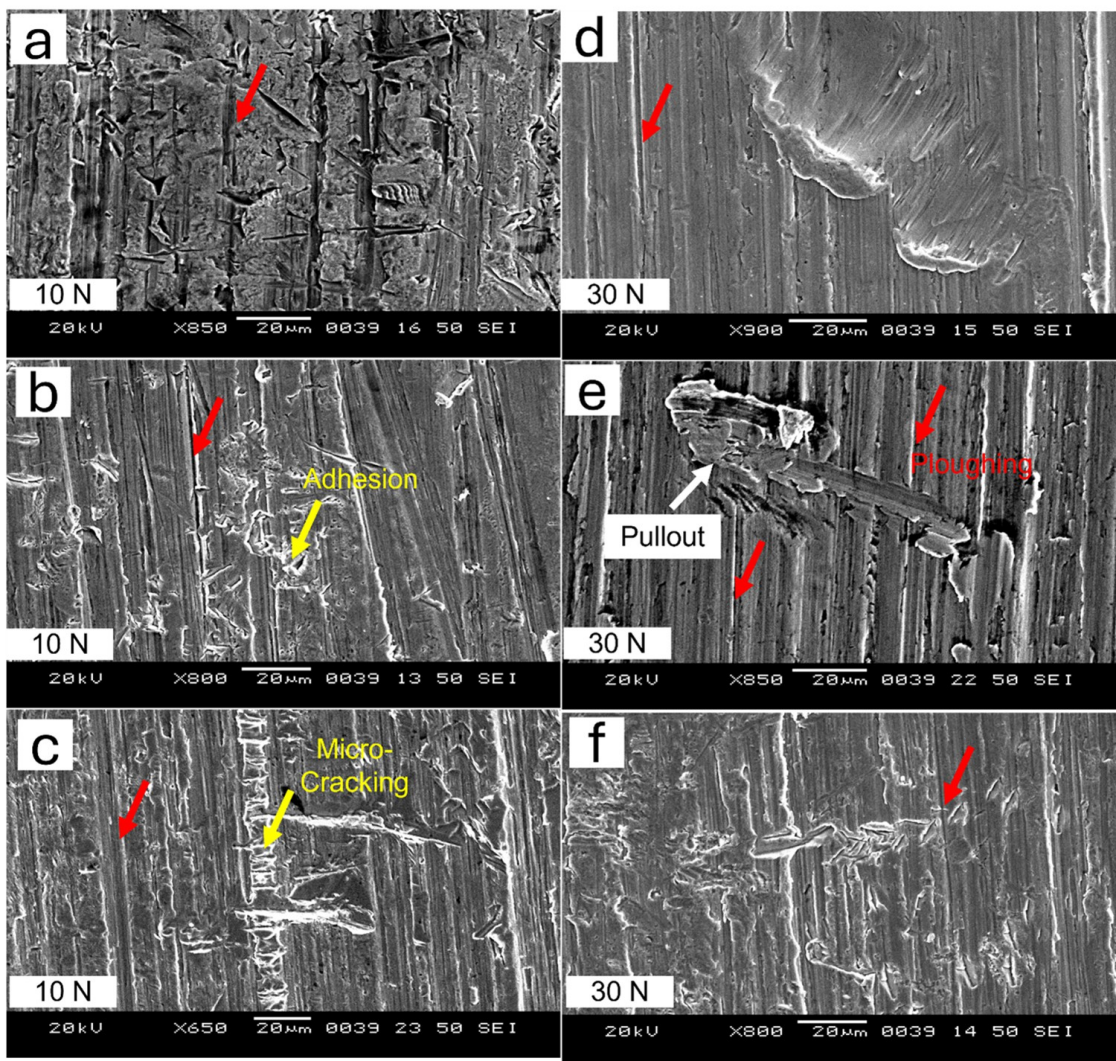
SEM images of the worn out surface of AISI420 in (a) as quenched (austenitized at 1198K for 45 min followed by quenching in oil) condition, and quenched followed by tempering at (b) 423K and (c) 873K at 10 N load, (d) as quenched, (e) 423K and (f) 873K at 30 N.

SEM analysis of the worn surfaces of the samples under specific conditions was carried out to investigate the micro-mechanisms of abrasive wear. The SEM micrographs of the worn surfaces of as-quenched and quenched-and-tempered MSS samples, subjected to loads of 10 N and 30 N, are shown in Figs 10 and 11 for AISI 410 and AISI 420 MSS, respectively.

In both the steel samples, (AISI 410 and AISI 420) many mechanisms of wear are identified and described as main reasons of material degradation such as microcracking, ploughing, groove formation, adhesion, or pullout of the particulate material (see Figs 10 and 11). By microcracking is meant the appearance of small cracks either on the surface of the hardened matrix or inside it, which occurs first and foremost due to the application of the mechanical force stress or due to thermal cycle. Eventually such cracks weaken the structure of the material making it over the period more and more susceptible to wear. This type of cracking usually starts in areas where there is high-stress concentration or regions with temperature variations, causing the material to become more fatigued than normal.

Ploughing is another important way of cutting, which incorporates excessive contact pressure or load, and hard particles move across the surface of the material. The grooves caused by this form of abrasive wear are associated with hard materials that contact the surface of the material during inertia movement producing deep grooves. In both the samples (AISI 410 and AISI 420), this groove formation highlights the role of proper lubrication in reducing friction-induced wear.

Additionally, adhesion emerges as a significant wear mechanism, particularly in the AISI 420 sample following 423K tempering. Adhesion in this context refers to the material transfer that happens when interacting surfaces stay together. Poor lubrication, high temperatures, and material compatibility all have a significant impact on this occurrence. Moreover, Fig 11E illustrates particle pullout, which can be attributed to fatigue wear or brittle fracture. Small material pieces separate from the surface as a result of this action, weakening the steel’s overall structural integrity. The cumulative effect of these wear mechanisms highlights how intricately mechanical stress, lubricant conditions, and material characteristics interact to determine how AISI 410 and 420 samples wear.

**Fig 12:**
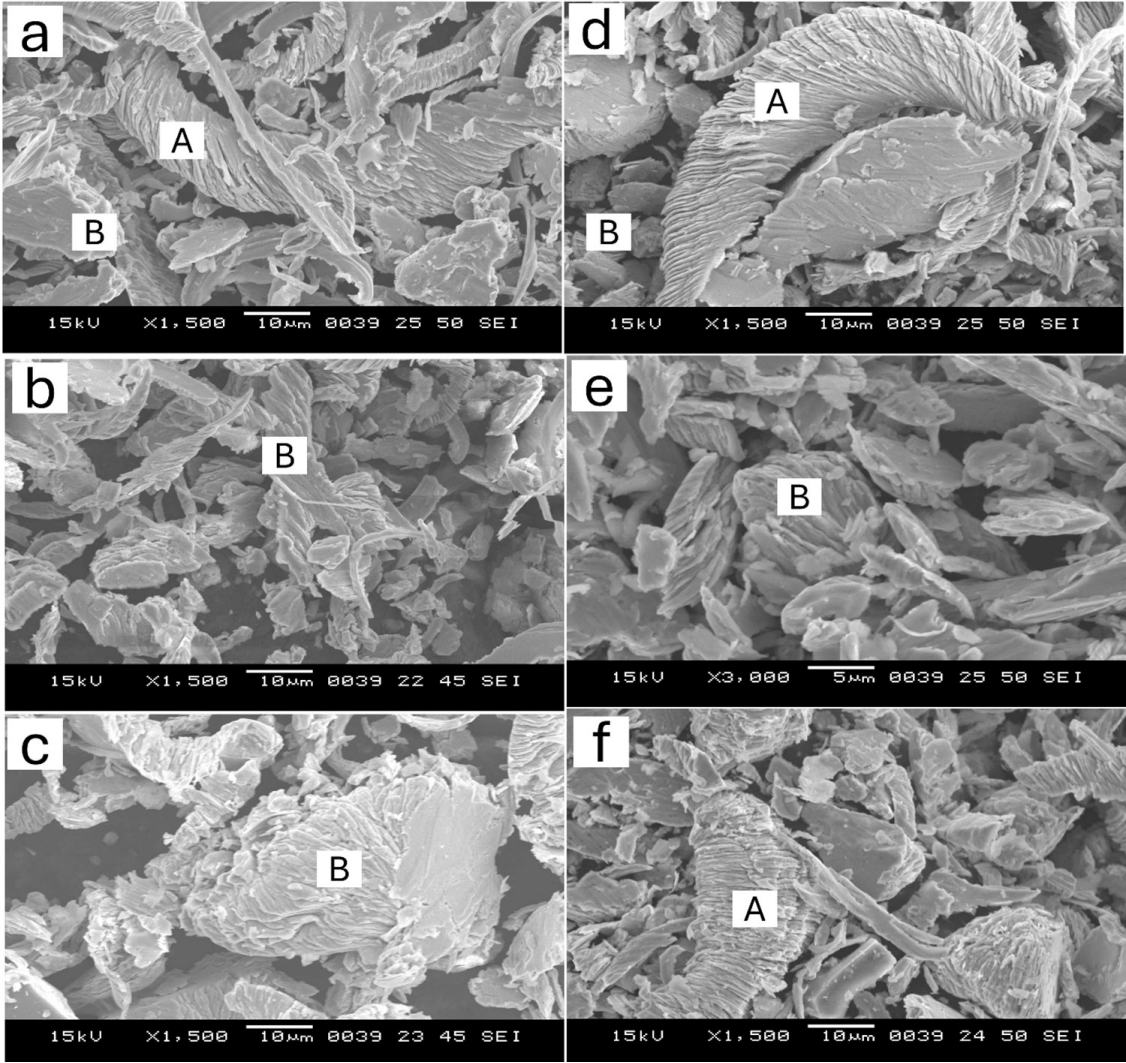
SEM of wear debris of AISI 410 steel in (a) as quenched (austenitized at 1198K for 45 min followed by quenching in oil) condition, and quenching followed by tempering at (b) 423K and (c) 873K at 10 N load, (d) as quenched, (e) 423K and (f) 873K at 30 N load (A: machining chips, B: flaky debris).

**Fig 13:**
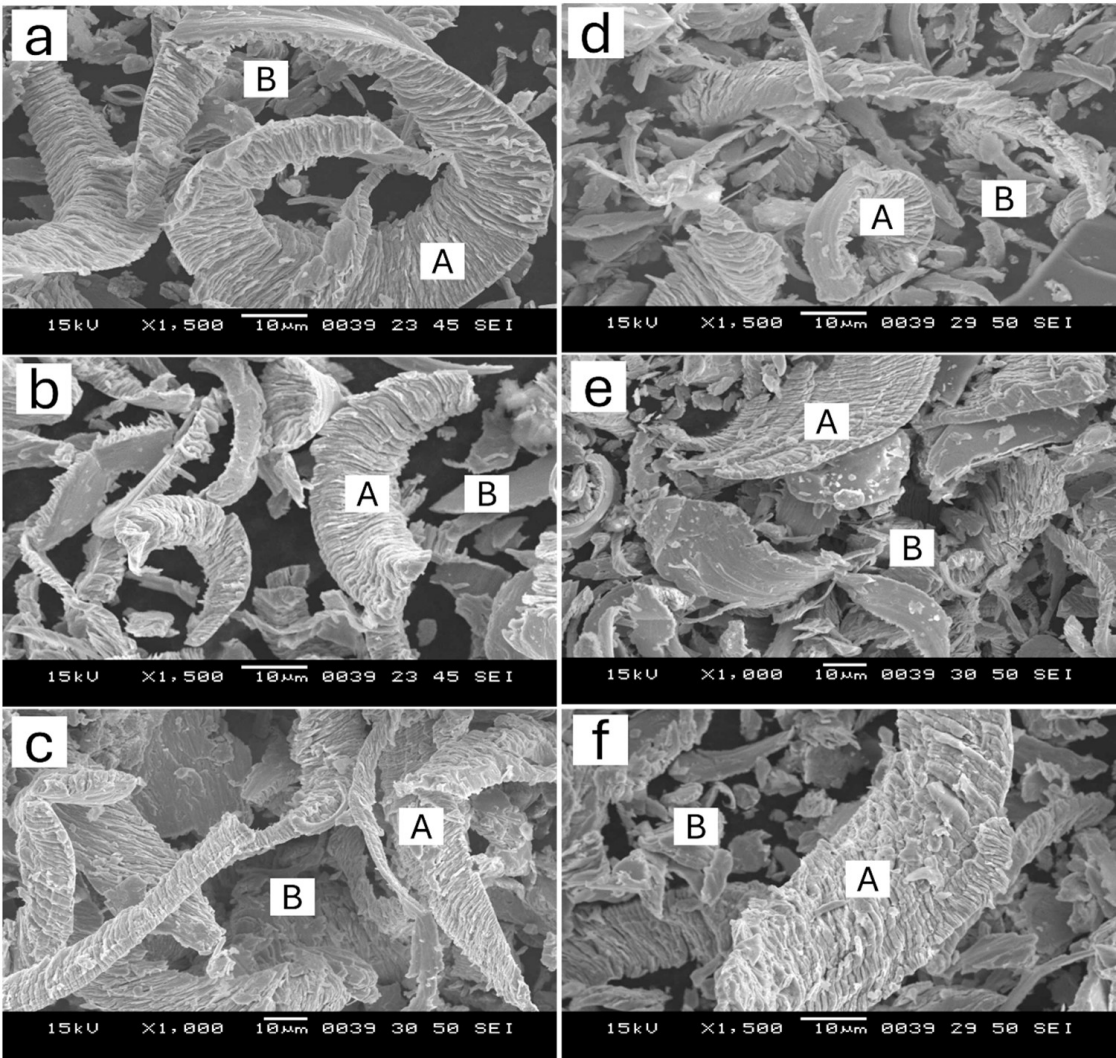
SEM of wear debris of AISI 420 steel in (a) as quenched (austenitized at 1198K for 45 min followed by quenching in oil) condition, and quenching followed by tempering at (b) 423K and (c) 873K at 10 N load, (d) as quenched, (e) 423K and (f) 873K at 30 N load (A: machining chips, B: flaky debris).

The wear was observed less in AISI 410 MSS samples than AISI 420 MSS samples. The mechanisms were more dominant at higher load in both the steels. At lower loads micro-groove formation, microcracking and micro-ploughing was the dominant mechanisms, while at higher loads multi directional groove formation along with ploughing and pull out of particles was more pronounced. The material removal was more in case of AISI 420 MSS since the matrix was weaker because of the precipitation of carbides. The carbon was taken out in the form of carbides making the martensite weaker resulting in more wear mass loss. SEM micrographs of the wear debris for selected condition is shown in Figs 12 and 13. The wear debris morphology supplements the SEM results of worn-out surfaces. Morphology difference in wear debris between AISI 420 and AISI 410 steel during wear testing can be explained with their distinct microstructural and compositional characteristics. AISI 420 steel generally has a higher carbon content in comparison to AISI 410, leading to increased hardness and wear resistance. AISI 420 having higher hardness results in a wear mechanism that favors the generation of more machining chips rather than flaky debris. These machining chips are indicative of a higher abrasive wear, where the harder material tends to cut or plow through the surface rather than deforming it plastically [2].

However, AISI 410 steel having low carbon content and relatively softer matrix, is more prone to plastic deformation during wear. Due to this, deformation results in the formation of more flaky debris as the material layers are gradually peeled off under the sliding contact. The softer nature of AISI 410 allows for more extensive material smearing and layering, leading to flake formation. So, the chip formation and flaky debris can be explained by the difference in carbon content and resultant hardness between these two types of steel (AISI 410, 420) significantly influences their wear debris morphology. In conclusion, difference in carbon content between AISI 410 and AISI 420 steels significantly affects their wear behavior. AISI 420, with higher hardness, generates more machining chips due to abrasive wear, while AISI 410, being softer, produces flaky debris from plastic deformation.

All samples show finer wear debris at a lower load of 10 N, indicating that higher loads produce coarser wear particles. The debris characteristics are also influenced by the load factor; higher loads result in greater wear and more noticeable debris generation, whereas lower loads cause less severe wear and finer debris.

## 4. Conclusions

Present work demonstrates the microstructures of AISI 410 and AISI 420 martensitic stainless steels after hardening and tempering were studied using optical and scanning electron microscopy. The effects of bulk hardness and microstructures on the two-body abrasive wear behavior were investigated. The worn-out surfaces and wear debris particles were also characterized using scanning electron microscopy to find out the mechanism of wear. Secondary hardening was observed at tempering temperature of 723 K. This was mainly due to the formation of (Fe,Cr)_23_C_6_. Martensite formation occurs after hardening. The undissolved carbides that remained coarsened after tempering (423 K) at low temperature. At higher tempering temperatures (873K) hardness was drastically decreased. Abrasive wear resistance of heat-treated martensitic stainless steel did not show any significant correlation with bulk hardness. AISI 410 showed lower wear mass loss compared to AISI 420 steel. The different abrasive wear mechanism includes micro-cracking, ploughing, groove formation, adhesion, and particle pullout. It was observed that a finer carbide morphology contributed to increased resistance to abrasive wear. The wear debris consisted of machining chips and flaky debris, which were relatively finer under lower loads.
